# Mineralization of Phosphorylated Fish Skin Collagen/Mangosteen Scaffolds as Potential Materials for Bone Tissue Regeneration

**DOI:** 10.3390/molecules26102899

**Published:** 2021-05-13

**Authors:** Eduardo P. Milan, Murilo Á. V. Rodrigues, Virginia C. A. Martins, Ana M. G. Plepis, Thomas Fuhrmann-Lieker, Marilia M. Horn

**Affiliations:** 1 Interunits Graduate Program in Bioengineering (EESC/FMRP/IQSC), University of São Paulo (USP), São Carlos 13560-970, Brazil; epmilan@usp.br (E.P.M.); amplepis@iqsc.usp.br (A.M.G.P.); 2 Physical Chemistry of Nanomaterials, Institute of Chemistry and Center for Interdisciplinary Nanostructure Science and Technology (CINSaT), University of Kassel, 34109 Kassel, Germany; th.fuhrmann@uni-kassel.de; 3 São Carlos Institute of Chemistry, University of São Paulo (USP), São Carlos 13560-970, Brazil; murilovigilato@usp.br (M.Á.V.R.); virginia@iqsc.usp.br (V.C.A.M.)

**Keywords:** fish skin collagen, mangosteen, phosphorylation, mineralization

## Abstract

In this study, a potential hard tissue substitute was mimicked using collagen/mangosteen porous scaffolds. Collagen was extracted from Tilapia fish skin and mangosteen from the waste peel of the respective fruit. Sodium trimetaphosphate was used for the phosphorylation of these scaffolds to improve the nucleation sites for the mineralization process. Phosphate groups were incorporated in the collagen structure as confirmed by their attenuated total reflection Fourier transform infrared (ATR-FTIR) bands. The phosphorylation and mangosteen addition increased the thermal stability of the collagen triple helix structure, as demonstrated by differential scanning calorimetry (DSC) and thermogravimetry (TGA) characterizations. Mineralization was successfully achieved, and the presence of calcium phosphate was visualized by scanning electron microscopy (SEM). Nevertheless, the porous structure was maintained, which is an essential characteristic for the desired application. The deposited mineral was amorphous calcium phosphate, as confirmed by energy dispersive X-ray spectroscopy (EDX) results.

## 1. Introduction

Biomaterials are remarkable for their functionality and design to potentialize the regenerative capacity of the body, overcoming conventional treatment limitations, assisting tissue regeneration, and improving quality of life. The main purpose is to replace or enhance the function of damaged tissues during the regeneration process. An example of biomaterial application is the fabrication of a scaffold, a porous structure that can stimulate cellular adhesion, growth, and proliferation, enhancing tissue regeneration in the applied area [1]. One important research area is bone regeneration, in which biocompatible materials, especially natural polymers, are used to obtain scaffolds that support cell proliferation and are able to withstand mechanical solicitations. In addition to the biomimetic properties, biodegradability is also desirable [2].

Since bone is naturally an organic/inorganic hybrid compound, studies that focus on mimicking its structure are a current topic in the tissue regeneration field. The combination of polymers and ceramics is an established approach to produce a scaffold for bone tissue regeneration and mimic the required properties of the material, such as biocompatibility, nonimmunogenicity, interconnected porous structure, for bone regeneration [3].

The major organic component of bone is type I collagen, which has a characteristic triple-helix structure responsible for polymer properties, especially biological interactions. The typical amino acid sequence defines the polypeptide chains where the prevalence of proline and glycine residues is necessary to avoid the formation of harmful aggregates. On the inorganic composition, hydroxyapatite is the main component, a calcium phosphate compound. It is responsible for the mechanical strength of the bone, and its crystals make up 70% of the bone structure [4].

Nature has numerous collagen sources, including amphibians, swine, bovines, poultry, and other mammalian sources. Due to easy access and cost-effectiveness, swine and bovine are the most common collagen sources in the tissue engineering field. However, they have been related to allergic reactions and illness [5], yet their use is questioned due to religious, ethnic, and regulatory conditions [6].

Aquatic sources have shown an alternative to those limitations. Besides, during the processing of products obtained from the aquaculture industry, a large quantity of waste is generated, reflecting massive environmental pollution, and transforming it into biomaterial has an economic gain, turning a low-value byproduct into a value-added product. The fish source is thought to be safer and an attractive alternative to conventional collagen sources due to its low cost, availability, and being mainly composed of type I collagen [7].

Tilapia (*Oreochromis niloticus*) is an easily adaptable fish species with worldwide breeding, reaching more than 6 million tons production since 2018 [8]. Amino acid composition changes according to fish species, modifying the molecular structure and consequently physicochemical and rheological properties [9]. Fish collagen has lower gelation and denaturation temperature but a higher viscosity when compared with mammalian sources. Decellularized tilapia skin scaffolds have shown promising applications in tissue engineering, Lau et al. [10] obtained a biocompatible scaffold as a suitable material for bone regeneration, with high mechanical strength and slow degradation rate.

The deposition of calcium phosphate in scaffolds is usually performed following the in vivo mineralization method. Although the mechanism is not entirely known, studies suggest that the nucleation process occurs on the surface of anionic groups, creating supersaturation points, and orienting crystal nucleation [11] along the arrangement direction of collagen fibrils [12]. Nevertheless, there is a consensus that negatively charged groups in apatite formation are essential in the nucleation step, whereas Ca^2+^ ion adsorption triggers nucleation. Du et al. [13] demonstrated the importance of phosphorylated proteins of the extracellular matrix in the regulation of the mineralization process, as the introduction of phosphate groups serves as new nucleation sites for apatite formation inside the collagen fibers [13].

For that reason, the phosphorylation of collagen is an approach that could increase the calcium phosphate precipitation, as the anionic groups serve as a site for homogeneous nucleation of the apatite crystallites. This biomimetic process may, in part, recapitulate the function of phosphate groups in naturally occurring phosphoproteins [14].

Mangosteen (*Garcinia mangostana* L.) has been identified as a source of xanthones, a polyphenol with a prominent effect on human health [15]. In the peel and seed waste materials, the primary xanthone derivative, α-mangostin, is described as a compound with various pharmacological activities such as antidiabetic, antioxidizing, and anti-inflammatory [16]. Kresnoadi et al. [17] described the use of mangosteen peel extract in association with demineralized freeze-dried bovine bone xerograft to stimulate alveolar bone regeneration and to treat the inflammation process caused by a trauma in teeth extraction. The study correlates the function of the xanthones in the antioxidant process, as their presence can activate wound-healing hormones, showing an advantage to combine this phenolic compound in scaffolds for bone tissue regeneration.

The use of polyphenolic compounds associated with biopolymeric scaffolds can improve not only the properties mentioned before but also improve the crosslink between polymeric chains and, consequently, enhance degradation rate and mechanical properties. With the main of modulating functional properties, numerous chemical reactions and physical interactions are available. Hydrophobic interactions would involve the abundant hydroxyl groups in the polyphenols structure and hydrophobic sites of the substrate molecules. This interaction plays an essential role in the complexation of polyphenols and collagen [18].

According to our knowledge, a study focusing on phosphorylated collagen functionalized with mangosteen peel extract aiming at the mineralization process is not found in the literature. This paper describes the use of sodium trimetaphosphate (STMP) to phosphorylate collagen obtained from an aquatic waste source. The prepared scaffold was functionalized with mangosteen peel extract, and the in vitro mineralization process was performed to induce the hydroxyapatite formation on the scaffold.

## 2. Results and Discussion

The following results were obtained after the analysis of the samples obtained in this study. The scaffold labeled C corresponds to the collagen extracted from Tilapia fish skin and was used as a sample control. After the phosphorylation process, the corresponding sample was named CP. Similarly, the scaffolds containing the two different concentrations of mangosteen were denominated C10P and C30P. Finally, the samples CP25, C10P25, C30P25, C10P37, and C30P37 were obtained after the mineralization process. The number corresponds to the temperature at which the experiment was conducted. All the descriptions about the sample preparation are found in the Section 3.

### 2.1. Collagen SDS-Page

**Figure 1:**
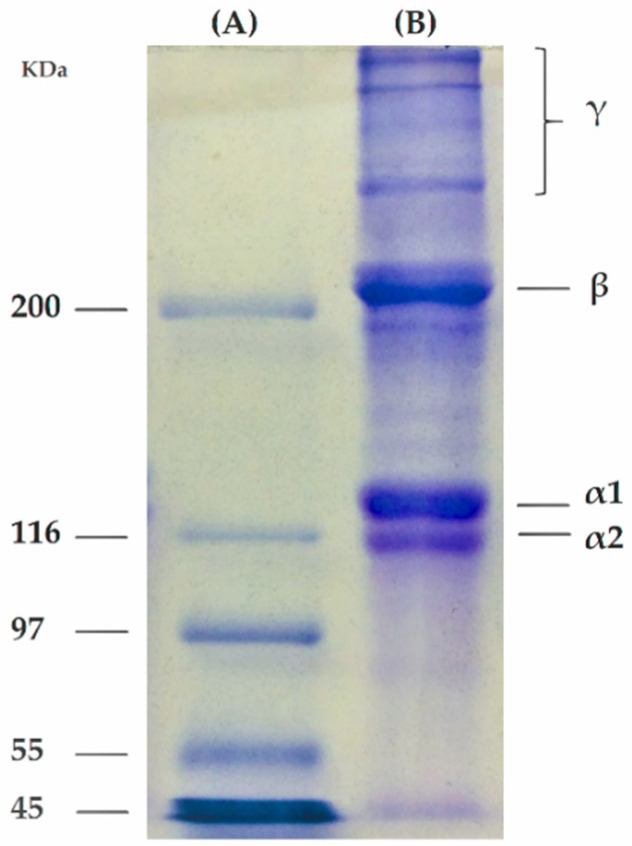
SDS-PAGE of (**A**) a protein marker; and (**B**) the extracted collagen from Tilapia skin.

The principle of SDS-PAGE is mixing the sample to the protein denaturant that contains SDS [19], and a bond is formed between the negatively charged SDS and the surface of the sample protein molecule. Depending on the size and shape of the protein chains, the movement through the gel is affected. The larger the molecular weight, the slower the movement. The bands formed on the gel allow the visualization and characterization of the collagen chains involved in forming the helix triple. The profile in Figure 1 is typical for type I collagen with intact alpha chains. 

Collagen extracted from tilapia fish skin mainly contained two identical α1 chains, one α2 chain and other less intense bands, β and γ bands, which can be associated with non-dissociated, respectively, dimers and trimers from α-helix and other crosslinked constituents [20]. The molecular weight of α chains extracted from fish skin was about 130–150 KDa, which can be originated from minor differences in amino acid composition influencing the molecular weight, being in accordance with the literature [21]. In addition, the α1/α2 ratio was measured with optical densitometry, and the value was 2.02, which indicates a trimer ((α1)_2_α2) that confirms a type I collagen.

### 2.2. Phosphate Determination

**Figure 2:**
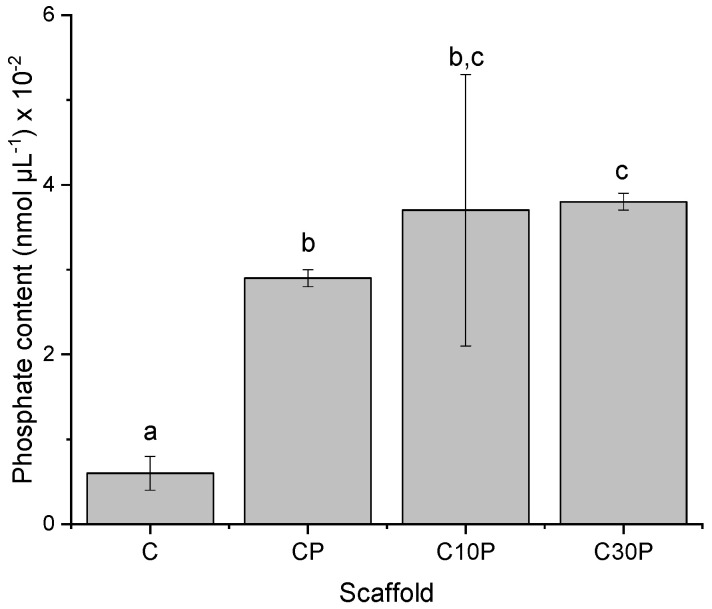
Quantitative comparison of phosphorous contents in phosphorylated scaffolds. (Same letter in the graphs means no significant difference; significance level of 5%).

Figure 2 shows the phosphorous content in the collagen scaffolds tested by phosphate colorimetric assay. 

The phosphorous content of non–phosphorylated collagen scaffold (C) was 0.6 10^−2^ nmol µL^−1^. Even though phosphate groups are not expected in this sample, similar behavior was described by Du et al. [13], in which a low amount of phosphate groups in non-mineralized rat tail collagen was found.

In the initial stages of mineralization, the phosphorylated groups in the collagen structure attract calcium ions electrostatically, improving its chelation. The direct introduction of phosphate groups in the polymeric structure increases the number of nucleation sites, which accelerates the initial nucleation process [22], and it is a feasible strategy to promote in vitro mineralization. 

Zheng et al. [23] described that hydrogen bonds and high conglutination might also increase nucleation sites. The role of the amorphous calcium phosphate nanoprecursors is to facilitate its infiltration into the collagen fibril gaps to promote mineral phase transformation from a parallel array into an oriented apatite crystal. These nanoprecursors are initially introduced as particles and only transform into apatite crystals before entering the collagen fibrils during biomimetic mineralization.

The content in phosphate groups was increased after the reaction with STMP, confirming that the phosphorylation process was successful. Interestingly, the mangosteen extract at the lower concentration showed a tendency to increase phosphate content compared to the phosphorylated scaffold (CP). Also, at the high extract concentration, there is a significant increase in that property. The increase in phosphate content related to extract concentration might be explained by the hydroxyl free groups of xanthones present in the extract that allow the phosphate incorporation by hydrogen bond interactions. As the samples were successfully phosphorylated, all were chosen in the following mineralization experiments.

### 2.3. ATR-FTIR

**Figure 3:**
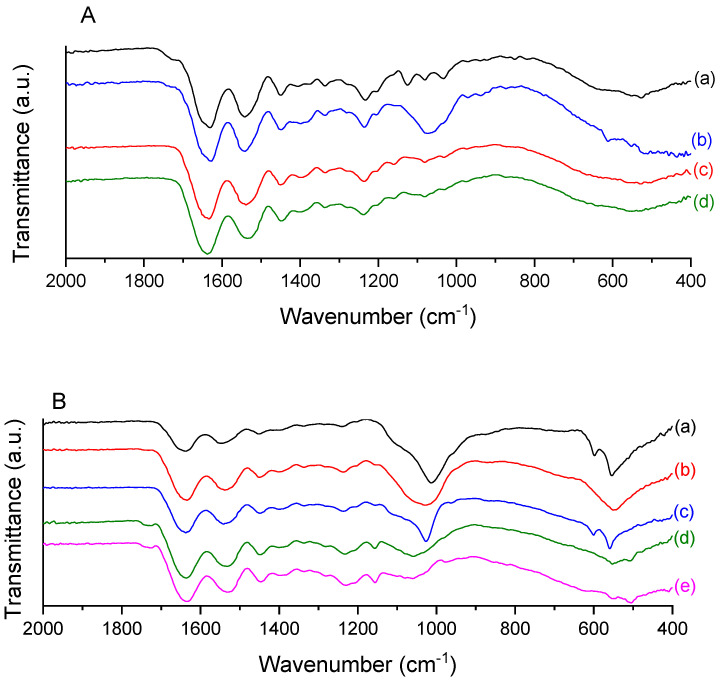
FTIR spectra of (**A**) collagen and phosphorylated collagen scaffolds: (a) C; (b) CP; (c) C10P; and (d) C30P, and (**B**) mineralized scaffolds: (a) CP25; (b) C10P25; (c) C10P37; (d) C30P25 and (e) C30P37.

The FTIR spectrum of the type I collagen from tilapia skin (C) is shown in Figure 3A. The typical amide bands can be observed, like amide A band in the 3272–3240 cm^−1^ region, amide B in the 2940–3080 cm^−1^ region, amide I between 1700–1600 cm^−1^, amide II around 1532–1555 cm^−1^, and amide III around 1240 cm^−1^. The described bands agree with the literature, confirming that the collagen extraction processes did not damage its structure. Amides I, II, and III bands are related to collagen structures and the presence of the collagen triple helix [24,25].

Phosphorylation of collagen was confirmed by FTIR spectra (Figure 3A). The bands around 1000 cm^−1^ indicate PO_4_^3−^ due to phosphate introduced by sodium trimetaphosphate (STMP). In the C and CP scaffolds, the phosphate band is visible and can distinguish from the others. Nevertheless, when the mangosteen extract is present, the intensity of the band is not so pronounced, which could be related to the interference with mangosteen as it also contains a band at around 1000 cm^−1^ that may hinder the PO_4_^3-^ signal visualization.

The spectra of the mineralized scaffolds (Figure 3B) show the phosphate bands at around 1015 cm^−1^, indicating the vibrational stretching of PO_4_^3−^ groups. In addition, the bands at around 560 and 600 cm^−1^ correspond to the deformational vibration that stands out related to phosphate salts formation, among them, hydroxyapatite [26], which is the target calcium phosphate form. The mineralized samples with 30% mangosteen (C30P25 and C30P37) do not show a distinctive 1015 cm^−1^ band, which might be due to interference of the high amount of extract. However, the second region mentioned before (around 560 and 600 cm^−1^) confirms the signs of PO_4_^3−^.

The collagen triple helix integrity is an essential property to be evaluated, as the absence of the helix means the rupture of the collagen structure. The ratio between 1240 and 1450 cm^−1^ bands is considered a method to evaluate the triple helix integrity. When the ratio is closer to 1, it implies collagen structural integrity, while a ratio close to 0.5 means the rupture of triple helix conformation into a randomly coiled form in which the three chains are separated [27,28]. Table 1 summarizes the ratio between the mentioned bands. The band at 1240 cm^−1^ is related to the amide III, and it is sensitive to structural changes in the collagen triple helix, and the band at 1450 cm^−1^ is associated with the proline and hydroxyproline C-H bond of pyrrolidine rings. 

As shown in Table 1, all the samples maintained the 1240/1450 cm^−1^ ratio close to 1, indicating that both phosphorylation and mineralization processes did not compromise collagen triple helix stability.

It is essential to maintain the protein stability, as its structure is crucial to provide biological interactions with cells and mimic the physiological conditions as close as possible.

**Figure 4:**
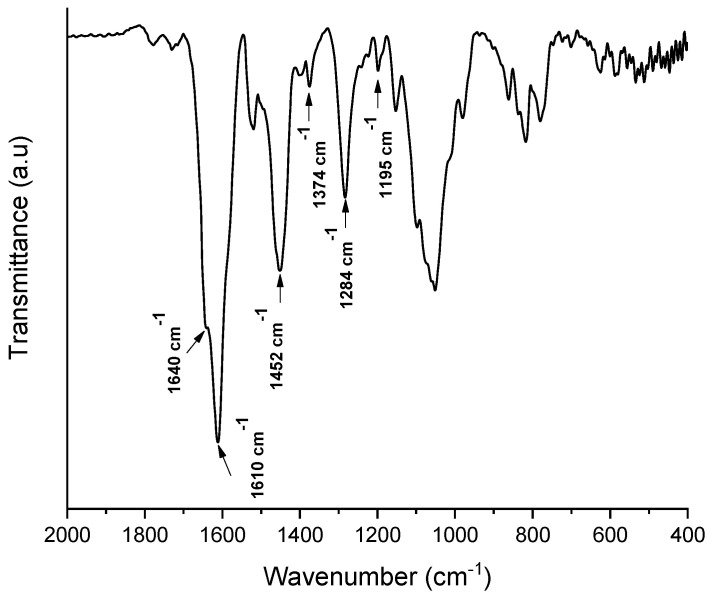
FTIR spectrum of the extract obtained from the mangosteen peel.

Figure 4 shows the spectrum of the mangosteen extract. Characteristics bands at 1450–1610 cm^−1^ from aromatic C=C, a band at 1640 cm^−1^ from C=O starching vibration of aromatic ketone, and 1450 cm^−1^ of skeletal vibration of C=C of aromatic hydrocarbon were observed. 

Additionally, bands at 1374 cm^−1^ refer to the symmetric bending vibration of -CH_3_, 1284 cm^−1^ to the C-O-C stretching vibration of -OCH_3_ on the benzene ring, and 1195 cm^−1^ to the stretching vibration of C-O-C. It confirms that the extract obtained from the mangosteen peel contains high quantities of xanthones and is very similar to the mains found xanthones, like α-mangostin [29].

### 2.4. Differential Scanning Calorimetry (DSC)

Thermal denaturation of collagen consists in the transition of ordered helix structure into random coil structure, and it is characterized by a discontinuity at baseline that is proportional to the difference in the heat capacity before and after denaturation [30]. In this case, the inflection point of this discontinuity furnishes the denaturation temperature (Td), in which during the heating of the sample, the breaking of intramolecular bonds in the collagen occurs, unwinding the triple helix. A higher Td indicates a stronger bonding within the collagen triple helix chains. Fish collagen is known to have lower Td than mammalian sources [31], so the increase of Td is desirable for most tissue engineering applications. 

**Figure 5:**
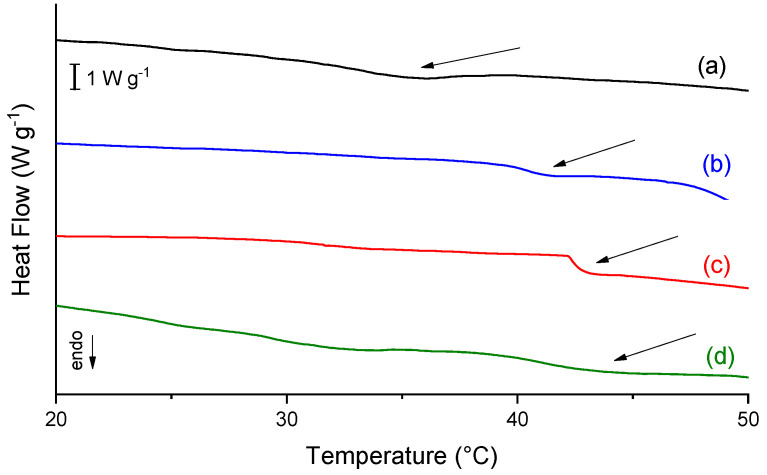
DSC curves of the scaffolds. In (a) C; (b) CP; (c) C10P; and (d) C30P.

Figure 5 shows the DSC curves in which the arrows indicate the thermal event of denaturation temperature. For collagen samples, it is usual that this transition is associated with a low heat flow [30,32,33]. Due to a large amount of calcium phosphates in the mineralized scaffolds, it is not possible to obtain the denaturation temperature, as its presence disturbs the observation of the thermal event.

As shown in Table 2, the phosphorylation process and mangosteen extract addition increase the Td, but showed no difference between them, and this property is independent of extract concentration.

Collagen stability is affected by the molecule composition and the post-translational modifications, molecular organization, and environment [32]. The increase of phosphate in samples contributes to a hydrophilic character in the samples, enabling an inter and intramolecular binding with solvating water enlarging the hydration network surrounding the collagen molecule, and stabilizing the triple helix. The addition of mangosteen extract did not affect the collagen triple helix stability, enabling the use of that component in the scaffolds without losing that valuable collagen property.

### 2.5. Thermogravimetry (TGA)

The TGA provided information about the effects of phosphorylation, mineralization, and extract addition, quantifying the amount of calcium phosphate deposited in the scaffolds. As the porous scaffold is composed of organic material, the weight loss observed between 200 to 700 °C was attributed to the decomposition and carbonization of that component. The residue at 700 °C refers to the inorganic component of the scaffold, which means the calcium phosphate deposits were obtained by the in vitro mineralization process. 

**Figure 6:**
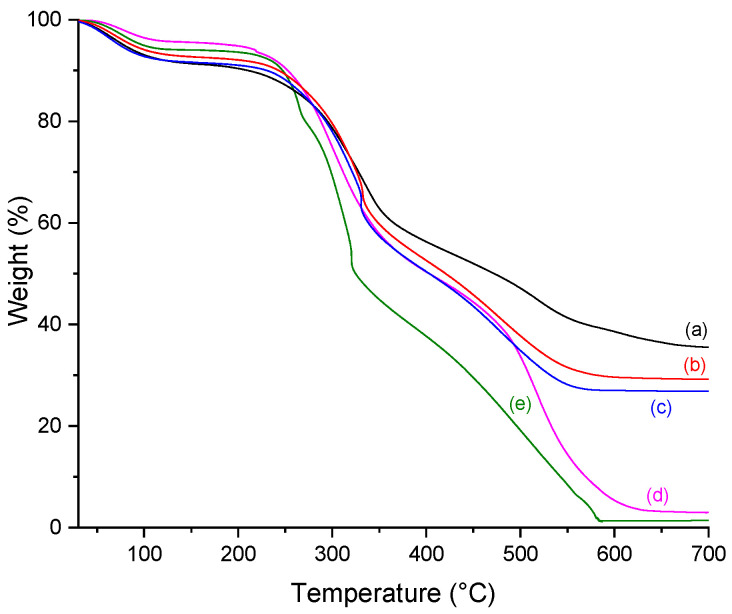
TGA curves for (a) CP25, (b) C10P25; (c) C10P37; (d) CP; and (e) C10P.

**Figure 7:**
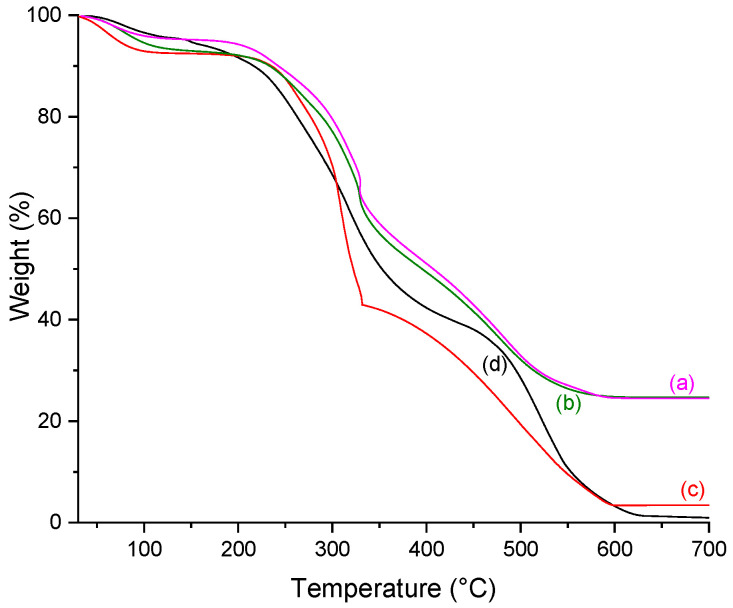
TGA curves for (a) C30P37; (b) C30P25; (c) C30P; and (d) C.

Thermal behavior was characterized by three stages of weight loss (Figure 6 and Figure 7). The first step (30–200 °C) is related to breaking the inter and intramolecular hydrogen bonds and the loss of water and disrupting the collagen triple helix structure. The lower values of water content present in the phosphorylated samples may be related to the phosphate group. It is known that molecules based on phosphoric acid catalyze the dehydration of products of biological origin, such as starch or lignin [34]. The second weight loss step (200–500 °C) occurs due to the degradation of proteins polymeric chains, and the third (500–700 °C) is related to the combustion of the organic components. 

Table 3 summarizes the weight loss percentage at each step, the onset of scaffold degradation, and the residue after carbonization in each sample, which allows the quantification of the mineralization process.

All scaffolds showed the same pattern of weight loss percentage. The first step, which corresponds to the water loss in the material, showed a slight tendency to increase in both mineralized and phosphorylated samples. After the degradation (200–500 °C) and carbonization (500–700 °C) steps, the observed mass loss curves differences are residue is assigned to the amount of non-organic material, i.e., the calcium phosphate deposited in the scaffolds, which leads to a smaller loss of mass for mineralized samples than for non-mineralized.

Mineralization increases the amount of residue in the samples, with values ranging from 24.50 to 35.52%. Even though the calcium phosphate amount is similar in the scaffolds, a slight decrease is observed when a high mangosteen concentration is used in the sample preparation. The presence of the natural extract can probably disturb the infiltration of the particles inside the collagen fibrils. Nevertheless, this behavior does not mean that those samples are unviable as a potential material for bone regeneration. The observed residue obtained by TGA measurements agrees with the notice phosphate presence in FTIR and UV-Vis results.

Evaluation of scaffolds degradation showed that the lowest T_onset_ value comes from the CP sample, which means all modifications, such as mangosteen addition and mineralization process at both temperatures (25 and 37 °C), increased the thermal stability of collagen helix triple. Comparing the phosphorylated sample (CP) with the mineralized at 25 °C (CP25), T_onset_ values showed that the mineralization was positively correlated to the collagen helix′s thermal stability triple. Its behavior can be attributed to the formation of apatite crystals into the collagen fibril gaps, stabilizing the collagen structure. 

In general, the increase in mangosteen concentration reflects an enhancement in the decomposition temperature, except when mineralization is performed at 37 °C. In that case, the highest T_onset_ observed for C10P37 instead of C30P37 can be due to both high mangosteen concentration and temperature close to the decomposition temperature, which could influence collagen structure stability. Unlike mammalian sources, collagen extracted from fish skin is more sensitive to temperature changes, which may explain this unexpected result.

### 2.6. Scanning Electron Microscopy (SEM)

The presence of pores in the three-dimensional scaffolds is an essential characteristic for bone tissue materials as it is an essential route for the transport of oxygen and nutrients. Structural parameters like pore size, pore morphology, interconnectivity, and orientation must be tailored to mimic the bone properties.

**Figure 8:**
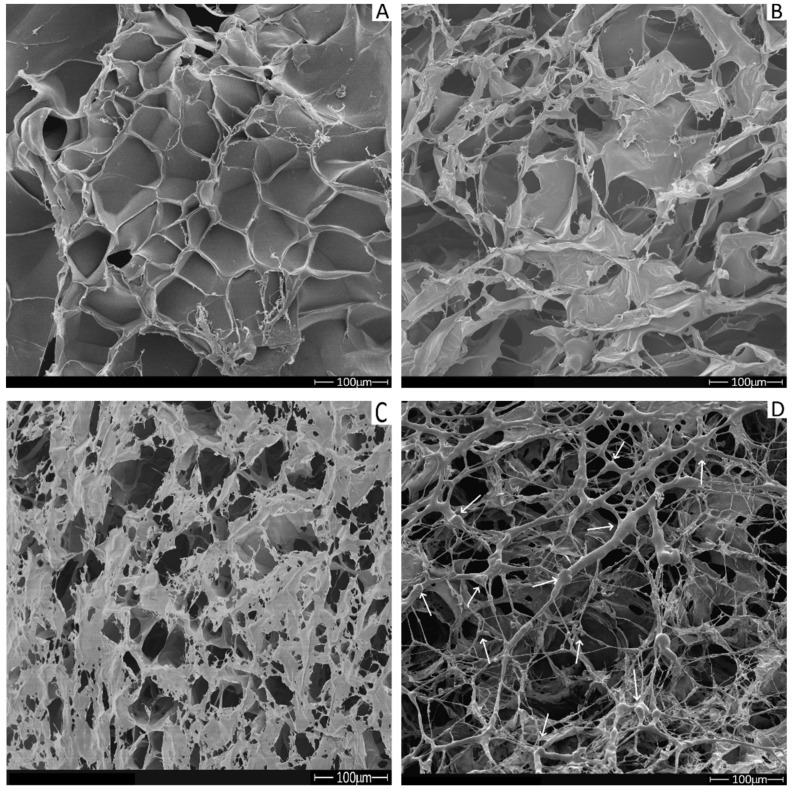
SEM Micrographs of collagen scaffold before and after phosphorylation. (**A**) C, (**B**) CP, (**C**) C10P, (**D**) C30P. Magnification of 200×.

Figure 8 shows the surface morphology of the scaffolds obtained by scanning electron microscopy (SEM), and the porous structure is visible in all samples. A significant advantage of this structure is that the pores are interconnected, an essential feature for conducting cells and vessels between pores. This condition fulfills cell growth and migration requirements for the target application [35].

The phosphorylation process did not change the surface morphology of the scaffold, as noticed when C and CP are compared (Figure 8A,B). Nevertheless, as shown in Figure 8C,D), there was a remarkable difference when the mangosteen extract is present in the scaffolds. More collagen filaments are observed on the surface, and it is directly related to the amount of the natural compound. In addition, a homogeneous extract distribution is visible in the C30P scaffold, as pointed by the white arrows (Figure 8D).

**Figure 9:**
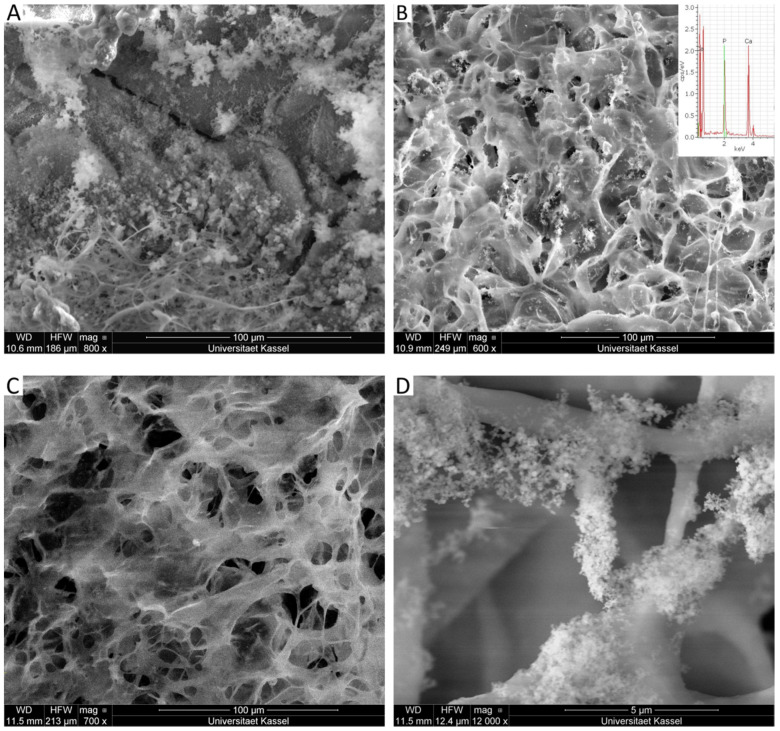
SEM Micrographs of collagen scaffold and calcium phosphate mineralization. (**A**) CP25; (**B**) C10P25, with respective EDX insert; (**C**) C30P25 (Magnification of 700×) and (**D**) C30P25 (Magnification of 12,000×).

An effective way to test the bioactivity and the ability to induce new bone tissue formation is the mineralization of the scaffolds. The presence of apatite crystals in the phosphorylated scaffolds along the surface of the collagen fibers was demonstrated in Figure 9. Despite that, scaffolds maintained their porous characteristic, an essential feature for cell attachment and growth. The extract induces a reduction of the crystals agglomeration (Figure 9B,C) with a sub-micrometer size (>100 nm) [36]. The presence of P and Ca is visible in the EDX inserted result, as shown in Figure 9B. A small crystal size reflects an increase in the internal surface, and it is positively related to an enhancement in proteins and cellular adhesion [37,38]. Moreover, a higher magnification SEM image (Figure 9D) demonstrated that the minerals were aggregates of spheroid crystals deposited on the collagen fibers.

In general, phosphorylation and mineralization reduce the average pore size, probably due to changes in the electrostatic interaction between collagen fibers associated with the presence of phosphate groups (Table 4). However, there were no noticeable changes in pore size after the extract addition, independent of mangosteen concentration. In other words, the presence of mangosteen does not affect the scaffolds′ porous size and does not change any electrostatic interaction between collagen fibers, as confirmed by DSC, with no differences in the thermal stability of the protein chains.

The apatite formation directly relates to the mean pore size, as after the mineralization process, a reduction in this feature was about 50% when the extract is found in the samples. A considerable decrease in pore size in the scaffold without the extract (CP and CP25) was noticed. It shows that the extract is an essential component in the scaffold composition as it keeps the size of the samples in a required range for the proposed application. This behavior is confirmed by TGA measurements, as the CP25 sample has a higher mineral content than CP, and the lower superficial pore size might be related to greater scaffold mineralization.

The pore size can be different for many functions due to cell size. For example, for the neovascularization process, a pore size of about 5 μm is necessary, while for bone tissue regeneration, a range between 100–350 μM is needed. In general, small pores (75–100 μM) allow osteoid tissue growth, and fibrous tissue penetration is possible when the pore size is between 10–75 μM [39].

The pore size does not affect the cell penetration depth or mineralization extent as even smaller pores allow osteoblast proliferation [40]. According to our findings, mineralized samples exhibit a small pore size. However, they can still participate in osteogenesis and other in vivo aspects, like the vascularization process [39].

### 2.7. Energy Dispersive X-Ray Spectroscopy (EDX)

Furthermore, EDX was also applied to explore the composition and structure of the deposited minerals. EDX measurements (Table 5) on the scaffolds demonstrated that the Ca/P ratio is in the amorphous calcium phosphates range between 1.2 and 2.2 [33]. However, in the human bone, this value ranges from 1.37 to 1.87.

All scaffolds showed a Ca/P ratio in the range typically found in the bone, which means that the mineralization process achieved its objective to deposit calcium phosphate to replace that tissue. Also, natural extract increases the calcium amount in the scaffolds, which must be related to possible complexation with the polyphenol structure of the mangosteen, especially its main component, α-mangosteen [41]. It is known that polyphenols increase mineralization and, consequently, bone formation by stimulating osteoblastic cells while inhibiting osteoclastic cells [42].

The phosphorylated collagen showed to be promising as a scaffold. Interestingly, the addition of mangosteen and the temperature at which the mineralization process was performed influenced the Ca/P ratio values. The obtained values are close to the theoretical hydroxyapatite (1.67) after the extraction addition and using the lower temperature (25 °C). It means that the mangosteen probably helps to stabilize the deposited calcium phosphate in the fibrils of collagen.

## 3. Materials and Methods

### 3.1. Materials

Fresh Nile Tilapia (*Oreochromis niloticus*) skins were randomly collected from the Pesque Pague Moinho (São Carlos, Brazil) and used as the raw material for collagen extraction. Mangosteen fruit was obtained at a local market (São Carlos, Brazil). All other reagents were of analytical grade and used as received without any purification process.

### 3.2. Extraction of Collagen

The fish skin was cleaned by scraping, removing scales, and residual meat, followed by washing in 0.9 wt. % saline solution to remove non-collagenous proteins on the surface. After that, it was rinsed thoroughly with deionized water. Fat contents were removed using ethanol: acetone 1:1 mixture as described by [31]. Afterward, the skins were exhaustively washed with deionized water to prepare for collagen extraction.

Collagen was extracted from Tilapia skin by alkaline hydrolysis according to [30] method, with slight modifications. Summarily, fish skin was immersed in a base solution (OH^−^ concentration 0.1 mol L^−1^) for 48 h, then in a sodium, potassium, and calcium sulfates and chlorides solution for 6 h to allow collagen triple helix stabilization. Excess salt was removed by successive rinsing steps of 1% boric acid, deionized water, EDTA 0.1%, and deionized water. Collagen was extracted with acetic acid pH 3.5, neutralized with sodium hydroxide, dialyzed against deionized water, and freeze-dried. Ultimately, the final material was crushed to obtain powdered collagen.

### 3.3. Mangosteen Extraction 

Mangosteen extract was obtained from fruit peels. The peels were manually removed, washed in water, dried, milled, and sieved at 500 µM. The obtained powder was refluxed at 50 °C in a methanol/ethanol 70/30 for 4 h [43]. After, the mixture was filtrated, and the solution was evaporated at 40 °C, and freeze-dried.

### 3.4. Phosphorylation of Collagen Scaffold 

Collagen solution with a concentration of 3% (*w*/*w*) was prepared by dissolving the freeze-dried fish collagen in 0.5% (*w*/*w*) lactic acid, and mangosteen extract was added in 10% and 30% according to collagen weight. Collagen scaffolds phosphorylation was performed using sodium trimetaphosphate (STMP), following the process [44,45], with slight modifications. Firstly, collagen scaffolds were suspended in 0.5% phosphoric acid (H_3_PO_4_) under constant stirring for 12 h, and the pH was adjusted to 9 with Na_2_HPO_4_. Then, STMP was added to the previous suspension at 10% based on the collagen weight and stirred for 6 h. 

The resulting material was dialyzed against deionized water and freeze-dried to obtain phosphorylated collagen (indicated by suffix “P” samples CP, C10P, and C30P). Control scaffold (C) samples without both mangosteen and phosphorylation method was prepared at 3% (*w*/*w*) concentration in 0.5% (*w*/*w*) lactic acid.

### 3.5. Mineralization of Scaffolds 

The mineralization of the phosphorylated scaffolds was performed by the alternate immersion method [46] with slight modifications. Solutions of 0.067 mol L^−1^ CaCl_2_ and 0.04 mol L^−1^ Na_2_HPO_4_ solutions, both buffered with 0.05 mol L^−1^ Tris buffer (pH 7.4), were used in this process. Scaffolds were placed in 10 mL of calcium chloride for 30 min, followed by washes in deionized water and subsequent soaking in 10 mL of sodium phosphate solution for 30 min. All experiments were conducted at 25 and 37 °C. Samples were labeled as CP25, C10P25, C10P37, C30P25, and C30P37, respectively. The scaffold CP37 was not possible to obtain, as it solubilized during the mineralization process.

### 3.6. Characterization 

#### 3.6.1. Collagen SDS-PAGE

The extracted collagen was analyzed by electrophoresis using glyceraldehyde-3-phosphate dehydrogenase (36,000 g mol^−1^), egg albumin (45,000 g mol^−1^), glutamic dehydrogenase (55,000 g mol^−1^), bovine serum albumin (66,000 g mol^−1^), phosphorylase b (97,400 g mol^−1^), β-galactosidase (116,000 g mol^−1^), and myosin 200,000 g mol^−1^) as molecular weight standards. Coomassie brilliant blue R-250 0.1% was used to visualize the gel after the electrophoresis following the method described by [47] with slight modifications. Band intensity was evaluated by optical densitometry using ImageJ software to analyze the SDS-PAGE gel.

#### 3.6.2. Phosphate Quantification

The total content phosphate was measured using a colorimetric method described by Vaeth et al. [48], with slight modifications. It is based on the complex yellow formation between phosphate with vanadate and molybdate in acid solution and is an accurate quantitative method to determine phosphates in organic samples.

Scaffold samples were acid digested on fuming HNO_3_ under heating and constant water replacement to prevent total liquid loss until all acid was evaporated. The maximum amount of added water was 10 mL. Then, vanadium molybdate reagent (Sigma-Aldrich) was added to the scaffolds in a 1:2.5 (reagent/sample ratio). After 20 min reaction time, the formation of a yellow complex was observed. The photometric extinctions of the samples were then measured at the maximum absorbance of 410 nm in a Perkin Elmer, LAMBDA 900 UV/VIR NIR Spectrometer, and results interpolated in a calibration curve previously determined using known PO_4_^3−^ concentrations.

#### 3.6.3. Attenuated Total Reflectance Fourier Transform Infrared Spectroscopy (ATR-FTIR)

Attenuated Total Reflectance Fourier-transform Infrared spectroscopy is a quick and reliable method to analyze a chemical structure. Samples were analyzed in an ATR_FTIR Bruker Alpha Platinum-ATR on a 4000–400 cm^−1^ interval with 24 scans and a resolution of 4 cm^−1^.

#### 3.6.4. Differential Scanning Calorimetry (DSC)

The scaffold samples (10 mg) were weighed and hermetically sealed in aluminum pans. The thermal profiles of the samples were assessed using a Perkin-Elmer DSC 7 from 10 to 60 °C with a heating rate of 5 °C min^−1^ under a nitrogen atmosphere (90 mL min^−1^). An empty sealed pan was used as the reference. The collagen denaturation temperature was calculated from the inflection point of the thermal curve. The observation of that thermal transition means the integrity of the collagen triple helix structure was preserved [30].

#### 3.6.5. Thermogravimetric Analysis (TGA)

TGA was carried out using a Perkin-Elmer, Pyris Diamond TG/DTA. Heating was performed in a platinum crucible in synthetic airflow (90 mL min^−1^) at a rate of 10 °C min^−1^ from 30 to 700 °C. The sample weight was in the range of 9–10 mg. The residue at 700 °C determined the calcium phosphate content.

#### 3.6.6. Scanning Electron Microscopy (SEM)

Scaffold morphology was analyzed with SEM, and the changes of collagen scaffold morphology with phosphorylation and mineralization procedures were studied. Phosphorylated and non-phosphorylated samples were placed in stubs, metalized with a 12 nm platinum cover in a Polaron Range metallizer with 1.10^–1^ mbar pressure chamber and 20 mA, and analyzed in a Hitachi S-4000 at 9 kV. Mineralized samples were not coated and were put in stubs and analyzed in an FEI-Quanta 250 FEG at 20 kV from the Werkstoffe des Bauwesend und Bauchemie, Universität Kassel, Germany. The software Image J was used for the pore size measurement. 

#### 3.6.7. Energy Dispersive X-Ray Spectroscopy (EDX)

EDX analysis enabled a semi-quantitative measurement of sample composition to determine the Ca/P ratio. Mineralized samples were submitted to the measurement in an FEI-Quanta 250 FEG equipment from the Werkstoffe des Bauwesend und Bauchemie, Universität Kassel, Germany.

Differences in phosphate determination, pore sizes, and Ca/P of the scaffolds were analyzed using analysis of variance (ANOVA) followed by Tukey’s test on Origin software. The significance level was set at 5%.

## 4. Conclusions

This study demonstrated that phosphorylated collagen/mangosteen scaffolds could induce apatite deposits on collagen fibrils. Collagen and mangosteen were successfully extracted from waste sources. With these materials, it was possible to produce a porous scaffold with suitable thermal and morphological properties. The phosphorylation of collagen was performed to improve the prenuclation of calcium phosphate clusters for mineralized scaffolds. The porous morphological characteristic was maintained after the inorganic crystals′ deposition, which is an essential feature for the intended application. EDX confirmed that amorphous calcium phosphate was deposited in the scaffolds.

Even though mangosteen extract does not influence Td in phosphorylated samples and reduces total mineral content, it allows handling the samples at higher temperatures, which turns it into a viable option for future studies and applications in the tissue engineering field. The introduction of mangosteen antioxidant and anti-inflammatory properties in the scaffolds is highly desirable in tissue regeneration.

The obtained mineralized scaffolds proved to be viable potential materials for the bone tissue engineering field and a new approach in studies of phosphorylated fish collagen with mangosteen peel extract. Future studies are still desirable, especially in vivo, to evaluate the material response to cellular attachment and tissue regeneration.

## Figures and Tables

**Table 1:** FTIR band intensity ratio.

Scheme 1240.	1240/1450 cm^−1^ Ratio
C	0.96
CP	1.01
C10P	1.02
C30P	1.03
CP25	1.04
C10P25	1.04
C30P25	1.00
C10P37	1.01
C30P37	0.98

**Table 2:** Denaturation temperature (Td) for the scaffolds.

Scaffold	Denaturation Temperature (°C)
C	33.5
CP	40.4
C10P	41.2
C30P	41.1

**Table 3:** Percentage of weight loss at different temperature range, residue percentage, and T_onset_ of scaffolds, obtained through TGA.

Scaffold	% Weight Loss			%Residue (700 °C)	T_onset_ (°C)
	30–200 °C	200–500 °C	500–700 °C		
C	8.34	63.16	27.54	1.04	228.8
CP	5.16	61.10	30.78	2.97	240.4
C10P	6.20	74.73	17.69	1.38	245.0
C30P	7.81	72.60	16.93	3.70	250.4
CP25	9.60	43.25	11.63	35.52	247.8
C10P25	7.91	54.32	8.58	29.19	249.8
C30P25	7.88	59.97	7.48	24.68	255.2
C10P37	8.96	56.15	8.04	26.85	262.9
C30P37	5.72	61.30	8.47	24.50	259.9

**Table 4:** Scaffolds average pore size.

Scaffold	Average Pore Size (μM) ± SD
C	85.0 ± 7.4 ^a^
CP	54.3 ± 8.4 ^b^
C10P	54.9 ± 8.6 ^b^
C30P	52.0 ± 7.7 ^b^
CP25	5.9 ± 1.0 ^d^
C10P25	24.5 ± 6.6 ^c^
C30P25	21.1 ± 3.1 ^c^
C10P37	24.6 ± 3.3 ^c^
C30P37	21.5 ± 3.1 ^c^

Note: Same letter in the column means no significant difference; significance level of 5%.

**Table 5:** Ca/P ratio for biomineralized scaffolds.

Scaffold	Ca/P Ratio ± SD
CP25	1.53 ± 0.10 ^c^
C10P25	1.73 ± 0.07 ^b^
C30P25	1.74 ± 0.08 ^b^
C10P37	1.91 ± 0.04 ^a^
C30P37	1.73 ± 0.09 ^a,b^

Note: Same letter in the column means no significant difference; significance level of 5%.

## Data Availability

Data available on request from the authors.

## Sample Availability

Samples of the compounds are available from the authors.
